# Multiple Sclerosis Risk Variant HLA-DRB1*1501 Associates with High Expression of *DRB1* Gene in Different Human Populations

**DOI:** 10.1371/journal.pone.0029819

**Published:** 2012-01-13

**Authors:** Antonio Alcina, María del Mar Abad-Grau, María Fedetz, Guillermo Izquierdo, Miguel Lucas, Óscar Fernández, Dorothy Ndagire, Antonio Catalá-Rabasa, Agustín Ruiz, Javier Gayán, Concepción Delgado, Carmen Arnal, Fuencisla Matesanz

**Affiliations:** 1 Instituto de Parasitología y Biomedicina “López Neyra”, Consejo Superior de Investigaciones Científicas (IPBLN-CSIC), Granada, Spain; 2 Departamento de Lenguajes y Sistemas Informáticos, CITIC, Universidad de Granada, Granada, Spain; 3 Unidad de Esclerosis Múltiple, Hospital Virgen Macarena, Sevilla, Spain; 4 Servicio de Biología Molecular, Hospital Virgen Macarena, Sevilla, Spain; 5 Servicio de Neurología, Instituto de Neurociencias Clínicas, Hospital Carlos Haya, Málaga, Spain; 6 Departamento de Genómica Estructural, Neocodex, Sevilla, Spain; 7 Centro Regional de Transfusión Sanguínea Granada-Almería, Ganada, Spain; 8 Servicio de Neurología, Hospital Virgen de las Nieves, Granada, Spain; Instituto de Ciencia de Materiales de Madrid - Instituto de Biomedicina de Valencia, Spain

## Abstract

The human leukocyte antigen (HLA) DRB1*1501 has been consistently associated with multiple sclerosis (MS) in nearly all populations tested. This points to a specific antigen presentation as the pathogenic mechanism though this does not fully explain the disease association. The identification of expression quantitative trait loci (eQTL) for genes in the HLA locus poses the question of the role of gene expression in MS susceptibility. We analyzed the eQTLs in the HLA region with respect to MS-associated HLA-variants obtained from genome-wide association studies (GWAS). We found that the Tag of DRB1*1501, rs3135388 A allele, correlated with high expression of *DRB1*, *DRB5* and *DQB1* genes in a Caucasian population. In quantitative terms, the MS-risk AA genotype carriers of rs3135388 were associated with 15.7-, 5.2- and 8.3-fold higher expression of *DQB1*, *DRB5* and *DRB1*, respectively, than the non-risk GG carriers. The haplotype analysis of expression-associated variants in a Spanish MS cohort revealed that high expression of *DRB1* and *DQB1* alone did not contribute to the disease. However, in Caucasian, Asian and African American populations, the DRB1*1501 allele was always highly expressed. In other immune related diseases such as type 1 diabetes, inflammatory bowel disease, ulcerative colitis, asthma and IgA deficiency, the best GWAS-associated HLA SNPs were also eQTLs for different HLA Class II genes. Our data suggest that the *DR/DQ* expression levels, together with specific structural properties of alleles, seem to be the causal effect in MS and in other immunopathologies rather than specific antigen presentation alone.

## Introduction

Multiple sclerosis (MS) is a common inflammatory disorder of the central nervous system characterized by demyelination with axonal and neuronal degeneration [1]. The cause of MS is unknown; however, susceptibility to MS is thought to be conferred by a combination of genetic and environmental factors [2].

The human leukocyte antigen (HLA) exerts the largest genetic contribution to MS susceptibility but exactly how it alters the risk of developing MS is not yet fully understood [3], [4]. Association studies based first on serological typing and more recently on genome-wide association studies (GWAS) have been conducted for MS and other autoimmune diseases, and have identified specific HLA-DR/DQ genes. However, the remarkably strong linkage disequilibrium (LD) across the HLA region has hampered the unequivocal ascertainment of the primary disease-risk HLA gene. This Class II association has been mapped to the DRB5*0101- DRB1*1501-DQA1*0102- DQB1*0602 haplotype in the North European population [5]. These alleles are almost always present together in this population, making it impossible to distinguish the primary association. The mechanism for the strong LD in these HLA haplotypes has been shown to be consistent with a functional epistatic interaction between DRB1*1501 and DRB5*0101 alleles. This functional epistasis is associated with a milder form of experimental autoimmune encephalomyelitis (EAE) in mice [6].

On the other hand, association studies in African-American populations have suggested that the DRB1*1501 allele itself determines MS-associated susceptibility [7]. However, in other populations, the risk allele or haplotype is different or does not contain DRB1*1501 as in Sardinians where MS is associated with the DRB1*0301–DQA1*0501–DQB1*0201 and DRB1*0405–DQA1*0501–DQB1*0301 haplotypes [8], or in African–Brazilian MS patients where the strongest association was observed with DQB1*0602 rather than DRB1*1501 [9]. In Caucasians, heterogeneity at the *DRB1* locus has also been found with respect to MS risk [10]. In Canadian MS families it has been observed that some DRB1*1501 haplotypes determine susceptibility while others do not [11] and that *DRB1*, *DQA1* and *DQB1* alleles contribute to MS susceptibility through epistatic interactions suggesting haplotypic rather than allelic HLA association [12].

As *DRB1* alleles have different structural properties for antigen presentation according to their amino acid sequence, MS-HLA association has been used to support the concept that disease pathogenesis is the result of an autoimmune reaction, perhaps against myelin-related antigens in the restricting context of DRB1*1501 [13]. However, this structural theory alone does not fully explain the association study results in MS. The description of polymorphisms that alter HLA gene expression [14], identification of several cis-acting genetic variants on expression of HLA class II genes [15], [16] as well as the recent observation that vitamin D may influence DRB1*1501 expression via a vitamin D response element [17], makes it possible that association of HLA class II polymorphisms with MS may be related to the levels of gene expression to the same or a greater extent than restriction of antigen response.

Variation in gene transcription is important in mediating disease susceptibility. Gene transcript abundance might be modified by polymorphisms in regulatory elements. In particular, much of the variation in gene expression levels and alternative splicing can be inherited [18], [19]. Polymorphisms that affect the expression levels of a gene are most often found near the gene itself, especially near the transcription start site [20], [21]. In most studies of gene expression genetics, generally diverse individuals are genotyped at genetic markers that characterize most of the common DNA variants in the population and are also phenotyped by measuring the abundance of mRNA transcripts by microarrays and more recently RNA sequencing (RNA-Seq) [22]–[25]. These molecular phenotypes are then genetically mapped like any other quantitative trait, revealing quantitative trait loci (QTL). Most studies of gene expression have classified expression variations into cis- or trans-acting factors, according to the proximity of the variant to the gene.

The cell lines genotyped by the international HapMap Consortium represent an ideal resource for expression QTLs (eQTLs) analysis. These lymphoblastoid B-cell lines, representing 11 human populations have been genotyped with approximately 4 million SNPs for CEU (Caucasian), YRI (Black Africans), CHB (Han Chinese) and JPT (Japanese) populations and with 1.5 million SNPs for other populations as part of the third phase of the HapMap project [26]. These cell lines from seven populations have been used to study variation in gene expression levels by microarray [20], [21] and cell lines from YRI and CEU by RNA-Seq [24], [25].

Here we integrate genotypes of the HLA polymorphisms and expression data for the *DRB5*, *DRB1* and *DQB1* genes in relation to the MS-GWAS associated variants to determine the role of the SNPs that alter the expression of these genes in MS susceptibility.

## Results

### The same SNPs correlated with expression levels of *DRB1* gene in seven different human populations

We explored the association in cis of the HLA region SNPs from HapMap and *DRB1* gene expression in different human populations. We analyzed ILMN_1715169 (*DRB1*) probe data from array quantification (Illumina Human-6 v2 Expression BeadChip) using RNA samples from 689 lymphoblastoid cell lines from 7 HapMap III populations: 80 from Han Chinese in Beijing, China (CHB), 82 from Gujarati Indians in Houston, Texas (GIH), 82 from Japanese in Tokyo, Japan (JPT), 83 from Luhya in Webuye, Kenya (LWK), 45 from individuals of Mexican ancestry in Los Angeles (MEX), 108 from Yoruban in Ibadan, Nigeria (YRI) and 109 from Utah residents with Northern and Western European ancestry from the CEPH collection (CEU). These cell lines have been extensively genotyped by the HapMap project, and we used the genotype data for the HLA region from positions 29677984 to 33485635 of chromosome 6 (Build 36). The number of genotyped SNPs in this region is 11564 for CEU, 11278 for YRI, 11564 for CHB, 11448 for JPT, 5703 for MEX, 5880 for LWK and 5650 for GIH.

**Figure 1:**
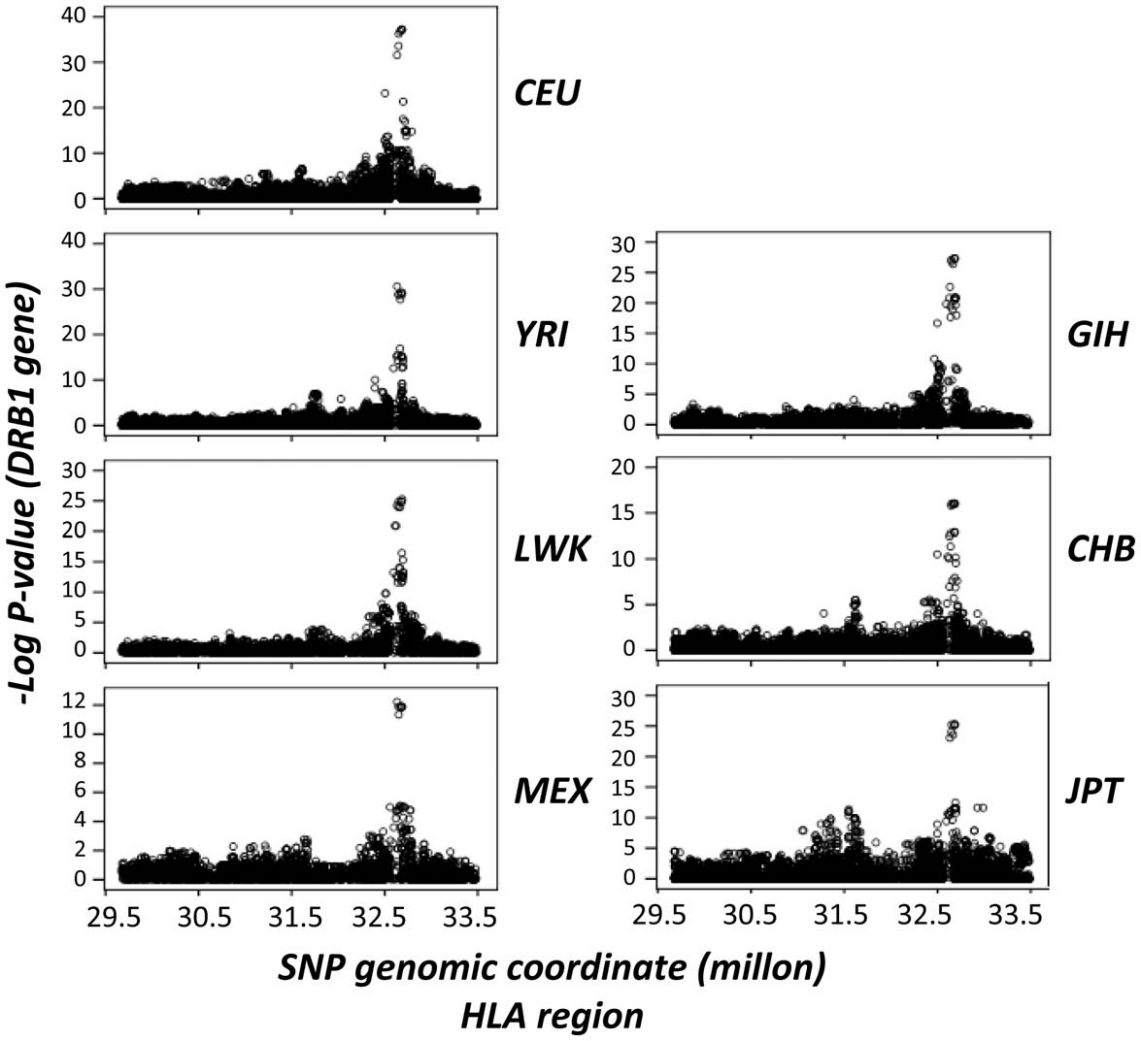
Association of HapMap III SNPs from the HLA region with expression levels of the HLA *DRB1* gene. The figure shows the strength of association between SNPs and gene expression levels, plotted as −log *P*-values. Coordinates are in NCBI Build 36. Shown are the CEU, YRI, LWK, MEX, GHI, CHB and JPT HapMap populations [26].

**Figure 2:**
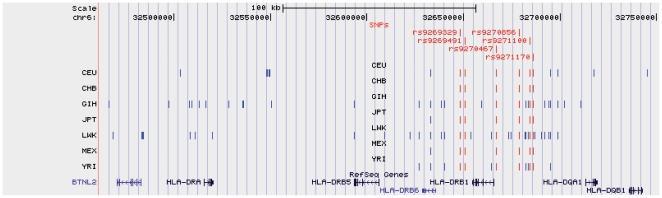
Location of SNPs associated with *DRB1* gene expression in the different human populations. Image from the UCSC browser showing the HLA Class II region. Vertical bars indicate the location of eQTLs (0.0001 permutation threshold) from all populations. Vertical red bars indicate the common eQTLs among all populations studied.

Spearman rank correlation (SRC) test for association between SNP genotypes and mRNA levels were performed as previously described [27] (Figure 1). Significant thresholds for each population were assigned through 10,000 permutations of expression values (Table S1). At the 0.0001 permutation threshold (PT), we discovered 21 CEU, 6 CHB, 33 GIH, 40 LWK, 7 MEX, 7 JPT and 14 YRI SNPs that were significantly cis-associated with *DRB1* expression. We observed that the most significant correlations corresponded to the same 6 SNPs in all human populations. Four SNPs were located upstream and 2 downstream of the *DRB1* gene (Figure 2). These 6 SNPs were in high LD in all the analyzed populations (r2 = 1−0.95). The minor allele frequency of these variants was similar in all populations, ranging from 0.31 in the CEU population to 0.17 the CHB population.

**Figure 3:**
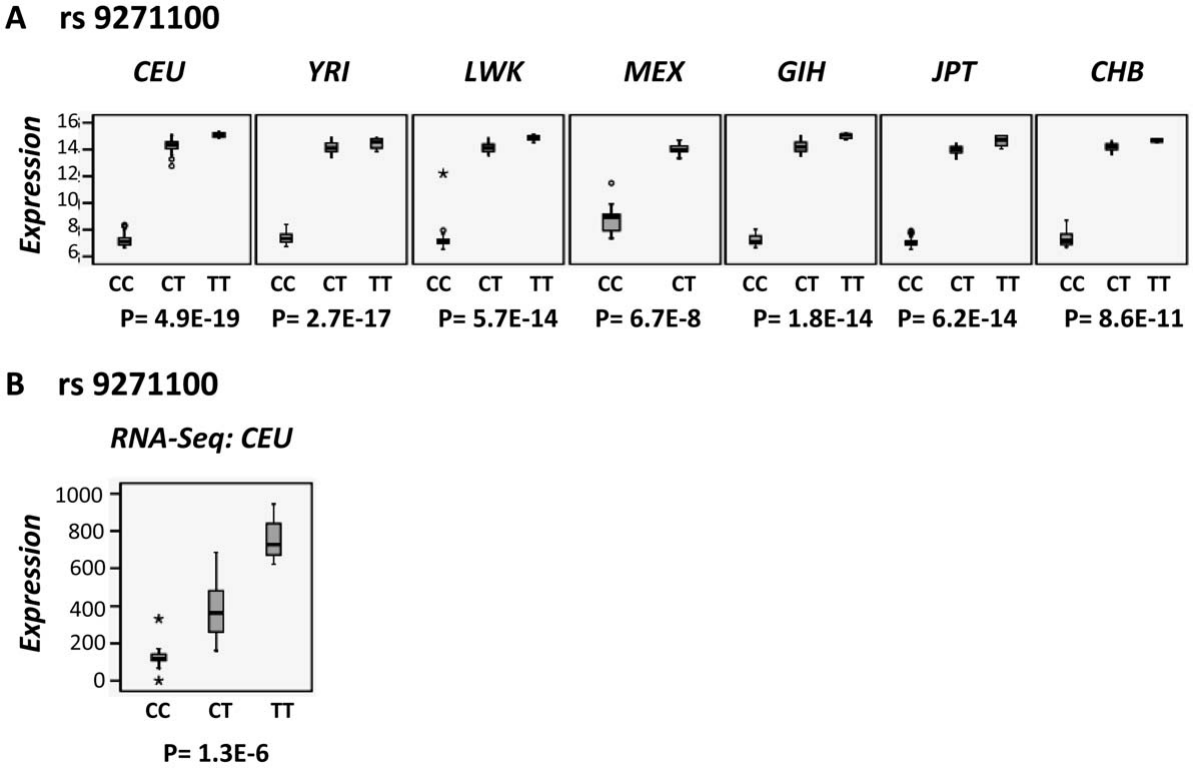
Association of the MS-risk variant rs9271100 with *DRB1* gene expression levels. In all plots, expression levels are represented for the three genotype groups. (**A**) Box plots of expression data from normalized results of ILMN_1715169 (*DRB1*) probe generated by Illumina Human-6 v2 Expression BeadChip (EMBL-EBI database (http://www.ebi.ac.uk/arrayexpress/) ID projects E-MTAB-198). (**B**) Box plot of expression data from the NM_002124 (*DRB1*) transcript of 41 CEU individuals obtained from RNA-Seq [19]. P-values are calculated by Kruskal Wallis Test.

After plotting the normalized expression of the *DRB1* gene by the genotypes of one of the 6 *DRB1*-expression associated SNPs (rs9271100), we observed a dominant effect of the T allele since individuals that carried TT or CT genotypes showed 1.6-fold higher expression (Figure 3 A). Almost no difference was observed between carrying 1 or 2 copies of the high expression allele. To confirm these data and rule out a possible artifact of the microarray technique, we plotted the expression data for the *DRB1* gene obtained from RNA-Seq of 41 CEU individuals [19] against the rs9271100 genotypes (Figure 3B). We observed, as with the microarray data, that the CC carriers express less *DRB1* mRNA than the CT and TT, but in this case the differences were larger, with a 3.3-fold increase on comparing CC with CT, and a 1.6- fold increase for TT with respect to CT, more consistent with an additive than dominant effect. The differences observed between both techniques could be due to an artifact, such as the signal for the CT and TT transcripts being above the signal saturation point of arrays.

We ruled out that the expression level differences described here were the product of the *Epstein-Barr* virus-cell line-immortalization since SNPs in LD with the ones described in this work have been reported as eQTLs of *DRB1*, *DRB5* and *DQB1* genes in RNA samples from postmortem liver tissues and liver surgical resections and monocytes [15], [28].

### The eQTLs for *DRB1* associated with MS susceptibility

Since *DRB1* gene is considered the primary association with MS in the HLA class II region, we evaluated whether the variants associated with the changes in mRNA levels of this gene were associated with MS susceptibility. We analyzed the data from the International Multiple Sclerosis Genetic Consortium (IMSGC) GWAS from 931 trios of Caucasian origin [29]. The GWAS analyzed 779 SNPs in the HLA region from 29671046 to 33473618 (build 36) positions of chromosome 6. The 779 SNPs do not include the 6 main eQTLs for *DRB1*. After crossing the eQTLs with the GWAS variants we observed an overlap of 3 SNPs between both lists. The following SNPs were the most strongly associated with MS in the GWAS: rs3129900 (P = 2.63E-34, OR 2.8, (2.3830–3.3350)), rs3129934 (P = 2.60E-37, OR 2.9 (2.4462–3.4048)) and rs9270986 (P = 2.38E-36, OR 3 (2.4938–3.5273)). These three SNPs were in high LD (r2 = 0.77−0.58) with the most significant eQTLs of *DRB1* gene. We also analyzed the most associated polymorphisms from the HLA region in other MS GWAS. For instance, the rs9271366 showed maximal association in two GWAS performed on Australia-New Zealand and Sardinian populations [30], [31]. This SNP is also an eQTL for *DRB1* gene.

### The most strongly associated MS variants were eQTLs for *DRB5*, *DRB1* and *DQB1* in the Caucasian population

**Figure 4:**
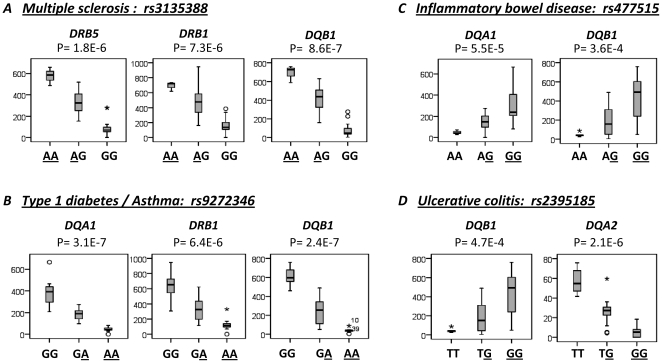
Association of risk-variants for different immune-related diseases with expression levels of HLA genes. Expression data are from CEU individuals obtained from RNA-Seq [9]. The data are from the transcripts *DQB1* NM_002123, *DQA1* NM_002122, *DRB1* NM_002124, *DQA2* NM_020056 and *DRB5* NM_002125. Underlined are marked the risk alleles.

We decided to analyze the eQTLs for other HLA genes with respect to MS association. For this purpose we used the eQTLs data generated by Mongomery et al. [24] by SRC testing for association between gene expression levels measured by RNA-Seq from 60 CEU HapMap samples with 1.2 million HapMap III SNPs. At 0.001 PT there were 11 genes with significant cis association, 4 HLA Class I *HLA-A*, *HLA-C*, *HLA-F*, *HLA-L* and 7 Class II *DRA*, *DRB5*, *DRB1*, *DQA1*, *DQB1*, *DQA2*, *DPB1*. We analyzed the overlap between the eQTLs of this region and the variants associated with MS by the IMSGC GWAS [29]. After crossing data from the IMSGC-GWAS and eQTLs, we obtained 41 eQTLs corresponding to 20 SNPs that were associated with MS with a P value<10^-7^. These were eQTLs for 6 genes, 1 HLA Class I, *HLA-C*, and 5 for HLA Class II, *DRA*, *DRB5*, *DRB1*, *DQA1*, *DQB1*. The most strongly MS-associated SNPs were eQTLs for *DRB5*, *DRB1* and *DQB1* simultaneously. This is due to the strong LD of the MS most associated variants with the eQTLs for the HLA genes. The rs3135388 SNP, tag for DRB1*1501 [32], the most associated variant with MS in the Caucasian population, was among the SNPs most associated with mRNA levels of *DRB5*, *DRB1* and *DQB1* simultaneously. We plotted the expression of these three genes, obtained from RNA-seq of 41 CEU individuals [19], with respect to the rs3135388 genotypes showing that the AA genotype, which is more frequent in the MS patients, correlated with higher expression of the three genes (Figure 4A). The homozygotic carrier for rs3135388 risk allele AA showed 15.7-, 5.2-, and 8.3-fold higher expression with respect to the GG carrier, and 1.6-, 1.5- and 1.8- fold higher expression with respect to the AG carrier for the *DQB1*, *DRB5* and *DRB1* genes, respectively.

To determine whether high expression of these three genes was associated with MS we genotyped the SNPs that best correlated with its expression in our MS Spanish cohort. These were rs9270986 for *DRB5* and rs9272346 for *DQB1*. The conditional logistic regression analysis with respect to the DRB1*1501 tag revealed that none of the variants contributed to the disease (Table 1). The haplotype formed by the alleles associated with high levels of expression of *DRB5*, *DRB1*, *DQB1* and DRB1*1501 allele had a higher OR than the DRB1*1501 alone (Table 2). DRB1*1501 was always in combination with high expression of *DRB5* and *DRB1*; therefore it was always highly expressed in the Spanish population. The haplotype formed by the *DRB1* and *DQB1* eQTLs, without DRB1*1501 allele, was not associated with disease, therefore the high expression of this two genes alone did not contribute to the disease.

**Table 1:** Conditional logistic regression analysis of the MS associated variants.

							*Conditional on*	
					*Unconditional*		*rs3135388*	
*Spanish*	*SNP*	*Risk allele*	*F_A*	*F_U*	*P*	*OR*	*P cond*	*OR*
eQTL DRB5	rs9270986	A	0.2451	0.1256	1.05E-22	2.392	0.117	1.396
eQTL DRB1	rs9271100	T	0.3481	0.2279	1.24E-17	1.881	0.01306	1.275
DRB1*1501	rs3135388	G	0.219	0.1031	1.96E-23	2.591	NA	NA
eQTL DQB1	rs9272346	G	0.4455	0.3917	0.0003381	1.265	0.356	0.9341

*African Americans*								
	rs9270986	A	0.2154	0.1898	0.15	1.173	0.74	1.043
	DRB1*1501	+	0.05705	0.03543	0.022	1.67	NA	NA

F_A, Frequency of affected; F_U, Frequency of unaffected; NA, not applicable; *P* cond, *P*-value of logistic regression analysis conditioned on the respective SNP; *OR*, odds ratio; *SNP*, single-nucleotide polymorphism. The data from African American are from McElroy et al. [7].

**Table 2:** Association with MS of the haplotypes bearing the DRB1*1501 SNPs and the eQTLs for *DRB5*, *DRB1* and *DQB1* genes.

*Population*	*Haplotypes*				*F_A*	*F_U*	*OR*	*P*
*Spanish*	*rs9270986*	*rs9271100*	*rs3135388*	*rs9272346*				
	**A** (>) *DRB5*	**T** (>) *DRB1*	***G*** DRB1*1501+	**G** (>) *DQB1*	0.2126	0.09729	2.67	1.93E-23
	**C** (<) *DRB5*	**T** (>) *DRB1*	***A*** DRB1*1501−	**G** (>) *DQB1*	0.1002	0.0977	1.03	0.777
	**C** (<) *DRB5*	**C** (<) *DRB1*	***A*** DRB1*1501−	**G** (>) *DQB1*	0.1195	0.1894	0.577	1.38E-09
	**C** (<) *DRB5*	**C** (<) *DRB1*	***A*** DRB1*1501−	**A** (<) *DQB1*	0.545	0.5984	0.799	0.000626

*African American*	*rs9270986*	DRB1*1501						
	**A** (>) *DRB1*	DRB1*1501+			0.05705	0.03357	1.77	0.0124
	**A** (>) *DRB1*	DRB1*1501−			0.1587	0.1567	1.02	0.891
	**C** (<) *DRB1*	DRB1*1501−			0.7842	0.8098	0.859	0.177

F_A, Frequency of affected; F_U Frequency of unaffected; OR , Odds Ratio; P, *P*-value of logistic regression analysis. Only haplotypes with higher frequency than 0.05 are shown. (>) stands for higher expression; (<) stands for lower expression. Data from African American are from McElroy et al. [7]. Haplotypes for African American are from rs9270986 (eQTL for *DRB5* and *DRB1* at the same time) and DRB1*1501.

### The DRB1*1501 allele is always highly expressed in the different human populations

Since the DRB1*1501 allele was always together with the *DRB1* eQTL in the Spanish population, we could not determine the contribution to MS of the DRB1*1501 allele in low expression conditions. To try to clarify this question we analyzed the association of these two variants in African American population which has a different LD in the region [7]. From the fine mapping of the HLA region performed by McElroy et al. [7] with 500 African Americans MS patients and 500 healthy controls, we obtained the genotype of the DRB1*1501 allele and the rs9270986 SNP, which in addition to the *DRB5* was eQTL for *DRB1* in all human populations (Table S1). We observed, as in the Spanish population, that the haplotype formed by the DRB1*1501 and the allele that correlated with high levels of expression of *DRB1* had a higher OR than the DRB1*1501 alone (Tables 1 and 2). However, the DRB1*1501, as in the Spanish population, was always in the same haplotype than the eQTL for *DRB1* and *DRB5*, making impossible distinguish the effect of the DRB1*1501 alone in this population as well (Table 2).

We investigated in other human populations if the DRB1*1501 and the eQTL for *DRB1* were also in the same haplotype as the one observed in the Spanish and the African American populations. For this end, we analyzed the haplotypes formed by the tag for DRB1*1501 and the rs9271100, eQTL for *DRB1* gene, in CHB, JPT and CEU populations. We observed that the T allele of rs9271100, associated with high expression of *DRB1* gene, was always together with DRB1*1501 (Figure S1).

### The most associated variants with other immunopathologies were also eQTLs for different HLA Class II gene combinations

**Figure 5:**
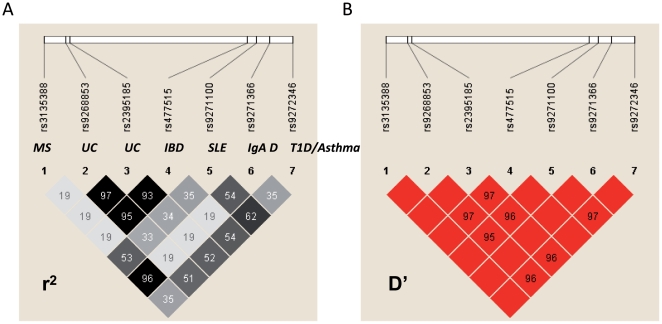
LD plots of the GWAS- SNPs associated with different immune related disease. Data are from HapMap III CEU population. (**A**) Linkage disequilibrium by r^2^. (**B**) Linkage disequilibrium by D′. Disease abbreviations: Type 1 diabetes (T1D), inflammatory bowel disease (IBD), ulcerative colitis (UC), systemic lupus erythematosus (SLE), asthma and IgA deficiency.

We decided to analyze whether the correlation between the expression of HLA Class II genes and HLA associated variants was specific for MS or could be extended to other autoimmune diseases. We obtained the GWAS results of several autoimmune and immune-related diseases in which a disease association with the Class II locus polymorphisms had been observed. We selected type 1 diabetes (T1D) [33], inflammatory bowel disease (IBD) [34], ulcerative colitis (UC) [35], [36], systemic lupus erythematosus (SLE) [37], rheumatoid arthritis (RA) [38], asthma [39] and IgA deficiency [40]. We analyzed the eQTLs for all HLA genes described by Mongomery et al. [24] with significance at 0.0001 PT with respect to the most associated SNP with the different diseases. In Table 3 we show the eQTLs that correlated with the SNPs indicating the specific transcript for which the correlation is strongest. We did not find any transcript of the RNA-Seq data that correlated with the most associated SNP with RA. Nonetheless, for the other diseases we found strong correlation with different combinations of genes of the HLA region. Figure 4 shows the expression of the different HLA genes with respect to the genotypes of the most associated variant for each disease. The associated allele correlated with higher expression of *DQB1* and *DQA1* for IBD, *DQB1* and *DQA1* for UC (Figure 4C, 4D) and *DRB1* for SLE as observed in MS. The other risk alleles for the diseases described in this work correlated with low levels of different genes (Figure 4B). The rs9271100 variant was described as associated with SLE in a Chinese Han population [37]. Although we did not have RNA-Seq data for this population, Figure 3 shows that TT carriers, homozygous for the risk allele, expressed higher levels of the *DRB1* gene in CHB population with microarray results. We observed that the variants associated with UC and IBD, as well as with MS and IgA deficiency, were in high LD, indicating a common origin (Figure 5). In MS and IgA deficiency, the susceptibility variants were in total LD. However, the risk allele in MS is associated with high expression while for IgA deficiency the risk allele is associated with low expression.

**Table 3:** Most associated HLA variants of different diseases and correlation with expression levels of HLA Class II genes.

Disease	SNP GWAS [ref.]	Gene	Transcripts	r Value	p Value	RiskAllele	Risk AlleleFrequency
**UC**	rs9268853 [35]	*DQA2*	ENST00000374940	0.728	4.33E-11	T	0.66
		*DQB1*	ENST00000399074	−0.637	4.41E-08		
	rs2395185 [36]	*DQA2*	ENST00000374940	0.728	4.33E-11	G	0.61
		*DQB1*	ENST00000399084	−0.637	4.41E-08		
**IBD**	rs477515 [34]	*DQB1*	ENST00000399082	0.591	6.50E-07	G	0.69
		*DQA1*	ENST00000374940	−0.708	2.41E-10		
**T1D**	rs9272346 [33]	*DQB1*	ENST00000399082	0.629	7.30E-08	G	0.61
		*DQB1*	ENST00000399074	0.809	6.00E-15		
		*DQB1*	ENST00000399084	0.85	<E-15		
		*DQA1*	ENST00000395364	0.803	1.20E-14		
		*DQA1*	ENST00000343139	0.769	7.27E-13		
**SLE**	rs9271100 [37]	*DRB1*	ENST00000360004	0.731	3.22E-11	T	0.14
		*DQB1*	ENST00000399084	0.621	1.17E-07		
**MS**	rs3135388 [29]	*DQB1*	ENST00000399065	−0.647	2.34E-08	A	0.22
		*DRB5*	ENST00000374975	−0.728	4.40E-11		
		*DRB1*	ENST00000360004	−0.702	3.98E-10		
**IgA deficiency**	rs9271366 [40]	*DQB1*	ENST00000399065	0.647	2.34E-08	A	0.85
		*DRB5*	ENST00000374975	0.728	4.40E-11		
		*DRB1*	ENST00000360004	0.702	3.98E-10		
**Asthma**	rs9272346 [39]	*DQB1*	ENST00000399082	0.629	7.30E-08	G	0.61
		*DQB1*	ENST00000399074	0.809	6.00E-15		
		*DQB1*	ENST00000399084	0.85	<E-15		
		*DQA1*	ENST00000395364	0.803	1.20E-14		
		*DQA1*	ENST00000343139	0.769	7.27E-13		

Diseases: Type 1 diabetes (T1D), inflammatory bowel disease (IBD), ulcerative colitis (UC), systemic lupus erythematosus (SLE), asthma, IgA deficiency and multiple sclerosis (MS). SNP GWAS indicate the most associated variant detected by GWAS, references in brackets. Transcripts are with Ensembl nomenclature. r values (correlation coefficient) and p-values from Spearman correlation test.

## Discussion

Our results indicate that the SNPs that have been extensively determined to be the main MS association signals, in the HLA Class II region, correlate with higher expression levels of *DRB5*, *DRB1* and *DQB1* genes. However, the association studies in MS Spanish and African American cohorts reveal that only changes in expression levels of these genes are not enough to explain the association. On the other hand, in all populations studied, due to LD, the DRB1*1501 allele correlate with higher expression of the *DRB1* gene. These data suggest that the high expression together with the structural characteristics of the DRB1*1501 receptor are, therefore, the real cause of MS association.

It has been shown that the immunological synapse strength in the interaction between the antigen–presenting cells (APC) and the T cell determine the fate into Th1 or Th2 type T cell [41]. The stronger TCR signal favor Th1 differentiation and this is dependent on the potency of TCR/peptide-MHC interactions, density of peptide-MHC complexes including co-stimulatory molecules and the duration of T cell-APC contacts. The high expression of Class II genes could affect directly to the concentration of peptide-MHC complex. In turn, a specific *DRB1* structure could affect on the duration and specificity of the interaction. Therefore, we propose that the higher o lower expression of the different HLA molecules open a spectrum of possible combinations with specific structure receptors and levels of co-stimulatory molecules that would determine the magnitude and quality of the T cell response, and the type of fate decision made by peripheral T cells. In fact, it has recently been determined by GWAS [30], [42] that polymorphisms close to co-stimulatory molecules such as CD40, CD86 and CD80 are also associated with MS.

In other autoimmune diseases, most of specific associated variants have in common that they are eQTLs for HLA genes. It is tentative to speculate that disease susceptibility lies in different combination of structural variants and gene level expression.

It is interesting to observe that in T1D the risk allele is associated with low DR and DQ genes expression but with high expression in MS, IBD, UC and SLE. It has been speculated that low levels of HLA molecules in the cortical thymic epithelial cells during thymic clonal deletion will generate a repertoire with increased self-reactivity T cells which in turn could contribute to the development of T cell-mediated autoimmunity [4]. The level of clonal deletion of autoreactive thymocytes is proportional to the concentration of MHC-self-antigen complexes in the thymus, which will depend on the levels of expression of the antigens and MHC receptors in the thymus.

One potential limitation of this study is regarding the complex nature of HLA, which is highly polimorphic and of repetitive nature. So, expression levels based on hybridization signals might yield false or spurious associations. This situation could be the case of some data of this study. However, to overcome this limitation, we analysed and reproduced array data with the data from RNA-Seq which is no based on hybridization specificity.

In conclusion, this work opens up a new understanding of the HLA disease associated variants in which the levels of expression of the DR and DQ could complement the structural properties for antigen presentation receptors. This would create additional levels of diversity and complexity when searching for the causal variants of the disease association in the HLA region.

## Materials and Methods

### Genotypes and gene expression data

We analyzed genotype and expression data from 689 unrelated individuals studied by the International HapMap project [26]. We used the HapMap Phase III genotype data, release #27. For *DRB1* gene expression, we used gene expression levels for the ILMN_1715169 probe that had been measured previously in lymphoblastoid cell lines from all 689 unrelated individuals using Array design A-MEXP-930 - Illumina Human-6 v2 Expression BeadChip [23]. We downloaded the data from EMBL-EBI database (http://www.ebi.ac.uk/arrayexpress/) ID projects E-MTAB-198 and E-MTAB-264. Data from RNA-Seq for *DRB1* (NM_002124), *DRB5* (NM_002125) and *DQB1*(NM_002123), DQA1 (NM_002122), *DQA2* (NM_020056) from 41 lymphoblastoid cell lines from CEU HapMap samples were generated by Cheung et al. and downloaded from (http://www.ebi.ac.uk/arrayexpress/) ID projects E-GEOD-16921 [19]. Data for eQTLs generated from 60 HapMap individuals of European descent were generated by Montgomery et al. [24] and downloaded from http://jungle.unige.ch/rnaseq_CEU60/.

### Expression association analysis and multiple test correction

We used Spearman's correlation to test for associations in *cis* between SNP genotypes and probe expression levels in each population. This method has been previously shown to produce robust results and avoids the effect of outliers in gene expression values [27]. We analyzed the SNPs from HapMap included between 29677984 to 33485635 of chromosome 6 (Build 36]. Significance thresholds for each gene were assigned after 10,000 permutations of expression values relative to genotypes. Computation of the Spearman correlation test has been performed with Genetranssoc (http://bios.ugr.es/Genetranassoc/), a c++ software with an analogous implementation of the Spearman coefficient in the statistical package R which also computes statistical significance using permutation tests.

### Study subjects

Case samples comprised 1049 Caucasian individuals with clinically defined MS according to Poser's criteria [43]. These samples were obtained from four public hospitals: the Hospital Clínico in Granada (n = 106), the Hospital Virgen de las Nieves in Granada (n = 145), the Hospital Carlos Haya in Málaga (n = 622) and the Hospital Virgen de la Macarena in Seville (n = 176). All three cities are within a 200 km radius in the South of Spain. The mean age of the individuals at case sample collection was 36 years and the mean age of controls at interview was 38 years. The percentage of females was 68% for cases and 68% for controls. All cases were classified as having RR (relapsing-remitting) or SP (secondary progressive) forms of MS. Controls were 972 blood donors with no history of inflammatory disease attending the blood banks. The study was approved by the Ethics Committees of Hospital Virgen Macarena, Sevilla, Hospital Virgen de las Nieves, Granada and Hospital Carlos Haya, Malaga, and written informed consent was obtained from all participants.

### SNPs Genotyping

rs3135388, rs9271100, rs9270986, rs9272346 were genotyped using Taqman® SNP Genotyping Assays (Applied Biosystems) methodology according to the manufacturer's specifications.

### Data management and statistical analysis

Association testing, Hardy–Weinberg Equilibrium, conditional logistic regression analyses, and haplotype analyses were carried out using PLINK version 1.07 [44]. Linkage disequilibrium patterns between SNPs were analyzed with Haploview 4.2 [45]. The Kruskal-Wallis test was performed with SPSS software.

## Supporting Information

Figure S1 **Haplotype formed by the tag for** DRB1*1501 **and the rs9271100, eQTL for** ***DRB1*** **gene, in CHB, JPT and CEU populations.** The LD plots and haplotypes are obtained from the HapMap data of the different populations. The DRB1*1501 allele is tagged by different allele combinations in each population as reported by Gregersen et al. (6). The Tags for DRB1*1501 are: CEU, rs3135388 G; CHB, rs7773756 T , rs6903608 C, rs620202 G; JPT, rs7773756 T, rs6919855 C, rs6901830 G.
(PDF)

Table S1 **Spearman correlation test of variants associated with DRB1 expression in different human populations.**
(XLSX)
