# Supplementary material for: Multiple Sclerosis Risk Variant HLA-DRB1*1501 Associates with High Expression of DRB1 Gene in Different Human Populations
Source: PLoS One. 2012 Jan 13;7(1):e29819. doi: 10.1371/journal.pone.0029819 (PMC3258250; doi:10.1371/journal.pone.0029819)
Supplement: Figure S1 — Haplotype formed by the tag for DRB1*1501 and the rs9271100, eQTL for DRB1 gene, in CHB, JPT and CEU populations. The LD plots and haplotypes are obtained from the HapMap data of the different populations. The DRB1*1501 allele is tagged by different allele combinations in each population as reported by Gregersen et al. (6). The Tags for DRB1*1501 are: CEU, rs3135388 G; CHB, rs7773756 T , rs6903608 C, rs620202 G; JPT, rs7773756 T, rs6919855 C, rs6901830 G. (PDF) [file pone.0029819.s001.pdf]

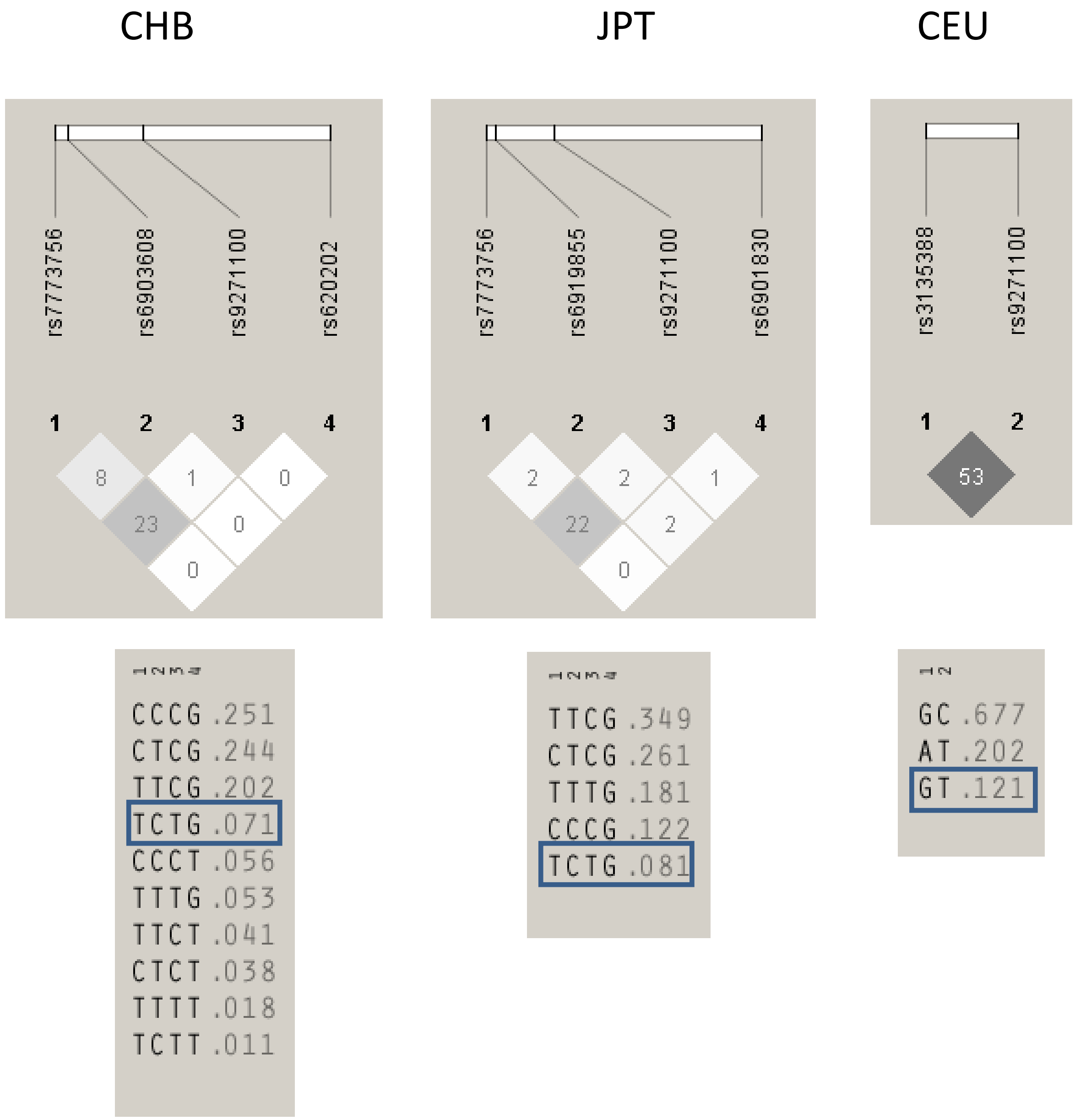
CHB
JPT
CEU
rs7773756
rs6903608
rs9271100
rs620202
1 2 3 4
8
1
0
23
0
0
rs7773756
rs6919855
rs9271100
rs6901830
1 2 3 4
2
2
1
22
2
0
rs3135388
rs9271100
1 2
53
1 2 3 4
CCCG .251
CTCG .244
TTCG .202
TCTG .071
CCCT .056
TTTG .053
TTCT .041
CTCT .038
TTTT .018
TCTT .011
1 2 3 4
TTCG .349
CTCG .261
TTTG .181
CCCG .122
TCTG .081
1 2
GC .677
AT .202
GT .121
